# Bayesian hierarchical models and prior elicitation for fitting psychometric functions

**DOI:** 10.3389/fncom.2023.1108311

**Published:** 2023-03-02

**Authors:** Maura Mezzetti, Colleen P. Ryan, Priscilla Balestrucci, Francesco Lacquaniti, Alessandro Moscatelli

**Affiliations:** ^1^Department Economics and Finance, University of Rome “Tor Vergata”, Rome, Italy; ^2^Department of Systems Medicine and Centre of Space Bio-Medicine, University of Rome “Tor Vergata”, Rome, Italy; ^3^Department of Neuromotor Physiology, Istituto di Ricovero e Cura a Carattere Scientifico Santa Lucia Foundation, Rome, Italy; ^4^Applied Cognitive Psychology, Ulm University, Ulm, Germany

**Keywords:** psychophysics, PSE, generalized linear mixed models, Bayesian model, psychometric functions

## Abstract

Our previous articles demonstrated how to analyze psychophysical data from a group of participants using generalized linear mixed models (GLMM) and two-level methods. The aim of this article is to revisit hierarchical models in a Bayesian framework. Bayesian models have been previously discussed for the analysis of psychometric functions although this approach is still seldom applied. The main advantage of using Bayesian models is that if the prior is informative, the uncertainty of the parameters is reduced through the combination of prior knowledge and the experimental data. Here, we evaluate uncertainties between and within participants through posterior distributions. To demonstrate the Bayesian approach, we re-analyzed data from two of our previous studies on the tactile discrimination of speed. We considered different methods to include *a priori* knowledge in the prior distribution, not only from the literature but also from previous experiments. A special type of Bayesian model, the power prior distribution, allowed us to modulate the weight of the prior, constructed from a first set of data, and use it to fit a second one. Bayesian models estimated the probability distributions of the parameters of interest that convey information about the effects of the experimental variables, their uncertainty, and the reliability of individual participants. We implemented these models using the software Just Another Gibbs Sampler (JAGS) that we interfaced with R with the package rjags. The Bayesian hierarchical model will provide a promising and powerful method for the analysis of psychometric functions in psychophysical experiments.

## 1. Introduction

Psychophysical methods are largely used in behavioral neuroscience to investigate the functional basis of perception in humans and other animals (Pelli and Farell, 1995). Using a model called the psychometric function, it is possible to test the quantitative relation between a physical property of the stimulus and its perceptual representation provided by the senses. This model has a typical sigmoid shape and relates the actual stimulus intensity (“physics”) on the abscissa to the probability of the response of the observer (i.e., perceptual response and “psychology”) on the ordinate, as collected with a forced-choice experiment. It is possible to summarize the performance of an observer by the parameters that are computed by the psychometric function: the Point of Subjective Equality (PSE), the slope, and the Just Noticeable Difference (JND) (Knoblauch and Maloney, 2012). The PSE estimates the accuracy of the response and corresponds to the stimulus value associated with a probability of response at chance level (*p* = 0.50). In two-interval forced-choice experiments, a deviation of the PSE from the value of the reference stimulus may indicate a bias, for example, in perceptual illusions (Moscatelli et al., 2016, 2019). The JND measures the noise of the response; the higher the JND, the higher the perceptual noise (Prins, 2016). The JND is an inverse function of the slope parameter of the psychometric function that is a measurement of the precision of the response. It is possible to test the slope or the JND of the function to evaluate the precision (or the noise) of the response.

Typically, generalized linear models (GLMs) are applied to estimate the parameters of the psychometric functions for each individual participant (Knoblauch and Maloney, 2012). In our previous study, we showed the advantages of using generalized linear mixed models (GLMMs) to estimate the responses of multiple participants at the population level (Moscatelli et al., 2012). A fairly comprehensive literature on fitting GLM and GLMM exists in different programming languages, including R, Python, and Matlab (Linares and López-Moliner, 2016; Schütt et al., 2016; Moscatelli and Balestrucci, 2017; Prins and Kingdom, 2018; Balestrucci et al., 2022).

In GLMM, we distinguish between fixed- and random-effect parameters (Stroup, 2012). The former, akin to the parameters of the psychometric function, estimates the effects of the experimental variables. Typically, the random-effect parameters estimate the variability across individual participants. In more complex data-sets, it is possible to account for other sources of unobserved variation by means of random-effect parameters. Blocking or batch effects are common examples of other random-effects parameters. The addition of this random component is the distinguishing feature of mixed models. For GLMMs, we assume that the random-effect parameters are normally distributed variables. The goal is to estimate the variance of that distribution. The larger the variance, the larger the heterogeneity across participants for a given parameter. However, the mean (or other central tendencies) of that distribution can be treated as if fixed effects have been applied to standard models. The conditional modes of the model estimating the response of individual participants can be treated as the fixed effects in standard psychometric functions. For example, in Balestrucci et al. (2022), we used conditional modes to plot the model estimates for individual participants.

A natural reinterpretation of the mixed model is the Bayesian approach, where all parameters are naturally considered as random variables, each having its own probability distribution (Zhao et al., 2006; Fong et al., 2010). Bayesian models provide not only a point estimate but also a probability distribution of the population parameter. Therefore, a Bayesian approach allows a natural assessment of the uncertainty in the parameter estimation. The advantages of the hierarchical Bayesian framework have been established in different fields in experimental psychology (Gelman et al., 1995; Rouder et al., 2003) and item response (Fox and Glas, 2001; Wang et al., 2002). To the best of our knowledge, only a few studies evaluate the use of Bayesian inference for fitting psychometric functions (Alcalá-Quintana and Garćıa-Pérez, 2004; Kuss et al., 2005; Schütt et al., 2016; Houpt and Bittner, 2018). In addition to estimating the intercept and the slope of the model, the flexibility of a Bayesian approach allows the study of uncertainties of the PSE.

This article is organized as follows. In Section 2, the two-stage Bayesian hierarchical model is proposed and discussed. Section 2.1 focuses on the description of prior distributions and Section 2.2 is dedicated to the discussion of the computational aspects. In Section 3, the data from two published experiments are considered. In Section 3.1, a Bayesian hierarchical model is fitted and compared with the results of Dallmann et al. (2015), while in Section 3.2, a Bayesian hierarchical model is fitted and compared with the results of Picconi et al. (2022). In Section 4, the two studies considered in Section 3 are jointly analyzed. Two alternative approaches are proposed. The first one considers the combination of the two studies with the parameters from the first study used as a prior distribution. The second approach introduces a parameter, *a*_0_, to quantify the uncertainty (or weight) of the first study that is considered as historical data—as detailed in Section 2.1. Finally, in Section 5, a discussion of the model is proposed and the results obtained are discussed.

## 2. Model

A typical data-set from a psychophysical experiment includes repeated responses from more than one participant. Fitting these types of data with ordinary generalized linear models (GLM) would produce invalid standard errors of the estimated parameters because they would treat the errors within the subject in the same manner as the errors between subjects. A viable approach to overcome this problem consists of applying a multilevel model (Morrone et al., 2005; Steele and Goldstein, 2006; Pariyadath and Eagleman, 2007; Johnston et al., 2008). First, the parameters of the psychometric function are estimated for each subject. Next, the individual estimates are pooled to perform the second-level analysis for statistical inference. Alternatively, it is possible to use the generalized linear mixed model (GLMM) that accounts separately for the experimental effects and the variability between participants using random- and fixed-effect parameters (Moscatelli et al., 2012).

Bayesian methods provide a viable solution for fitting models of the GLM and GLMM families (Gelman et al., 1995; Rouder and Lu, 2005). In particular, Kuss et al. (2005) have applied Bayesian methods for estimating a psychometric function, based on a binomial mixture model. A Bayesian hierarchical model is a statistical model written in multiple levels (hierarchical form) and estimates the parameters using Markov chain Monte Carlo (MCMC) sampling. Applying a Bayesian hierarchical model consists of the following processes: (i) model definition, including specification of parameters and prior distributions in different levels, (ii) update of the posterior distributions given the data, (iii) and Bayesian inference to analyze the parameters' posterior distributions (McElreath, 2020).

In the current study, we considered data from two-interval forced-choice discrimination tasks, as mentioned in the two example data-sets detailed in Sections 3.1 and 3.2. A two-stage Bayesian hierarchical model has been applied to these data-sets, with a probit model for each individual subject at the first stage. Let *X* denote the experimental variable (or variables), and let *Y* be the response variable that consists of binary responses. Thus *Y*_*ij*_ = 1 if subject *i* in trial *j* perceived a comparison stimulus with value *x*_*ij*_ as larger in magnitude (e.g., depending on the specific task, faster, stiffer, heavier, brighter, etc.) than a reference stimulus. As for the example data analyzed in this article (speed discrimination task), *Y*_*ij*_ = 1 if the subject perceived the comparison as “faster” than a reference one. The relationship between the response variable and the experimental variables is defined as:


(1) $$\begin{array}{lll} Y_{ij} & \sim & Bern\left(\mu_{ij}\right) \end{array}$$

(2) $$\begin{array}{lll} {\text{Φ}}^{-1}\left(\mu_{ij}\right) & = & \alpha_{i}+\beta_{i}x_{ij} \end{array}$$

The model assumed that the forced-choice responses *Y*_*ij*_ are independent and identically distributed (i.i.d.) conditional on the individual parameters (α_*i*_, β_*i*_). In case of repeated measurement, for each subject and conditions, Equation (1) can easily be substituted by


(3) $$\begin{array}{l} Y_{ij}\sim Binom\left(\mu_{ij},n_{ij}\right) \end{array}$$

where *Y*_*ij*_ represents, the number of “faster” responses for subject *i* at condition *x*_*ij*_.

The function Φ^−1^ in Equation (2) establishes a linear relationship between the response probability and the predictor that is fully described by two parameters α_*i*_ and β_*i*_. The probit link function Φ^−1^ is the inverse of the cumulative distribution function of the standard normal distribution *Z*. That is:


$$\begin{array}{l} \mu_{ij}=P\left(Z\leq \alpha_{i}+\beta_{i}x_{ij}\right)\;\forall i,j \end{array}$$

For more details on probit link function refer to Agresti (2002) and Moscatelli et al. (2012). Other link functions like Logit and Weibull are also often used in psychophysics (Agresti, 2002; Foster and Zychaluk, 2009).

In the first stage, the model characterized the behavior of each individual participant *i*. The second level defines the model across all participants, similar to the GLMM described by Moscatelli et al. (2012). To this end, the second level estimates the overall effects across subjects by combining individual-specific effects. The parameters (*a, b*) describe the overall model and results from the combination of the subject-specific parameters, taking into account their uncertainties. Through a Bayesian hierarchical approach, the second level takes into account the uncertainties of the subject-specific parameters. It assumes the following distributions:


(4) $$\begin{array}{lll} \alpha_{i} & \sim & Norm\left(a,\tau_{\alpha }\right) \end{array}$$

(5) $$\begin{array}{lll} \beta_{i} & \sim & Norm\left(b,\tau_{\beta }\right) \end{array}$$

(6) $$\begin{array}{lll} a & \sim & Norm\left(\mu_{a},\sigma_{a}\right) \end{array}$$

(7) $$\begin{array}{lll} b & \sim & Norm\left(\mu_{b},\sigma_{b}\right) \end{array}$$

Appropriate hyperprior distributions for (τ_α_, τ_β_, σ_*a*_, and σ_*b*_) need to be specified. The precision of the overall model and the between-subjects variability are gained by the posterior estimates of the parameters τ_α_ and τ_β_, respectively. In the application in Section 3.1, we will discuss different prior distributions for τ_α_ and τ_β_, which may be different for each subject or depend on other covariates. The proposed framework provides a reliable approach to account for the uncertainty of the fixed effects parameters.

The precision and the accuracy of the response are estimated by the parameters of the model. The slope parameters β_*i*_ link the inverse probit of the expected probability and the covariates *x* (i.e., the stimulus). Therefore, this parameter estimates the precision of the response, the higher is the estimated value of β_*i*_, the more precise is the response. The interpretation of the location parameter of the psychometric function depends on the nature of the psychophysical task. In forced-choice discrimination tasks, as mentioned in the two examples detailed in Sections 3.1 and 3.2, the PSE estimates the accuracy of the response. The response is accurate if the PSE is equal to the value of the reference stimulus. The value of the PSE relative to observer *i*, *pse*_*i*_ is computed from intercept and slope in Equation (2) as follows:


(8) $$\begin{array}{l} pse_{i}=-\frac{\alpha_{i}}{\beta_{i}} \end{array}$$

The PSE corresponds to the stimulus value yielding a response probability of 0.5, that is, the point at which participants are equally likely to choose the standard or the comparison stimulus in response to the task. In the examples mentioned later the PSE participants are equally likely to choose one stimulus or the other to the question “which stimulus moved faster?”.

### 2.1. Prior distribution

According to the Bayesian paradigm, prior distributions and likelihood constitute a whole decision model. Ideally, a prior distribution describes the degree of belief about the true model parameters held by the scientists. If empirical data are available, then new information can coherently be incorporated via statistical models, through Bayesian learning. This process begins by documenting the available expert knowledge and uncertainty. A subjective prior describes the informed opinion of the value of a parameter before the collection of data.

Prior distributions as described in the previous paragraph are non-informative prior distributions. The flexibility of the Bayesian model allows to modify (Equations 4, 5) by considering, for example, partition or group of subjects between historical and current data. We assume that there is one relevant historical study available. However, the approaches proposed here can in principle be extended to multiple historical studies. Here, we recall the method based on the power prior proposed by Ibrahim and Chen (2000). This has emerged as a useful class of informative priors for a variety of situations in which historical data are available (Eggleston et al., 2017).

The power prior is defined as follows Ibrahim and Chen (2000). Suppose we have two data-sets from the current study and from a previous study that is similar to the current one, labeled as the current and the historical data, respectively. The historical data are indicated as *D*_0_ = (*n*_0_, *y*_0_, *x*_0_), while the current data are indicated as *D* = (*n, y, x*), *n*, and *n*_0_ are the sample size, *y* and *y*_0_ are the response vectors, respectively *n* × 1 and *n*_0_ × 1 vectors. Finally, *x* and *x*_0_ are (either *n* × *p* matrix or *n*_0_ × *p* matrix ) the covariates. Let indicate θ as the vector of parameters, π_0_(θ) represents the initial prior distribution for θ before the historical data *D*_0_ are observed. The parameter *L*(θ|*D*) indicates a general likelihood function for an arbitrary model, such as for linear models, generalized linear model, random-effects model, non-linear model, or a survival model with censored data. Given the parameter *a*_0_, between 0 and 1, the power prior distribution of θ for the current study is defined as:


$$\begin{array}{l} \pi \left(\theta |D_{0},a_{0}\right)\propto L{\left(\theta |D_{0}\right)}^{a_{0}}\pi_{0}\left(\theta \right). \end{array}$$

This way, *a*_0_ represents the weights of the historical data relative to the likelihood of the current study. According to this definition, the parameter *a*_0_ represents the impact of the historical data on *L*(θ|*D*).

Depending on the agreement between the historical and current data, the historical data may be down-weighted, reducing the value of *a*_0_. The main question is what value of *a*_0_ to use in the analysis, which means how to assess agreement between historical and current data and how to incorporate the historical data into the analysis of a new study. The easiest solution is to establish a hierarchical power prior by specifying a proper prior distribution for *a*_0_. A uniform prior on *a*_0_ might be a good choice, or a more informative prior would be to take a Beta distribution with moderate to large parameters. Although a prior for *a*_0_ is attractive, it is much more computationally intensive than the *a*_0_ fixed case. The *a*_0_ random case has been extensively discussed (Ibrahim et al., 1999, 2015; Ibrahim and Chen, 2000; Chen and Ibrahim, 2006). Another approach, computationally more feasible, is to take *a*_0_ as fixed and elicit a specific value for it and conduct several sensitivity analyzes about this value or to take *a*_0_ as fixed and proceed, for example, with a model selection criterion.

### 2.2. Computational aspects

The large improvements in the availability of computational packages for implementing Bayesian analyzes have allowed the growth of applications of hierarchical Bayesian models. Many of the available packages permit the implementation of the Monte Carlo Markov Chain (MCMC) algorithm which saves time by avoiding technical coding. MCMC sampling is a simulation technique to generate samples from Markov chains that allow the reconstruction of the posterior distributions of the parameters. Once the posterior distributions are obtained, then the accurate and unbiased point estimates of model parameters are gained. Software for the application of Bayesian models is currently applied in several different fields (Palestro et al., 2018; Myers-Smith et al., 2019; Zhan et al., 2019; Dal'Bello and Izawa, 2021; Mezzetti et al., 2022). Gibbs sampling is an MCMC algorithm that can be implemented with the software Just Another Gibbs Sampler (JAGS), (Plummer, 2017). It is possible to interface JAGS with R using the CRAN package *rjags* developed by Plummer (2003). The reader may refer to the following tutorials for fitting hierarchical Bayesian models using JAGS (or STAN) and R (Plummer, 2003; Kruschke, 2014).

Once the model is defined in JAGS, it is possible to sample from the joint posterior distributions. The mean of samples from the posterior distribution of the parameters provides the posterior estimates of the parameters of interest. From the samples of the posterior distribution, it is also possible to extract the percentile and provide the corresponding 95% credible intervals.

As a diagnostic tool to assess whether the chains have converged to the posterior distribution, we use the statistic $\hat{R}$ (Gelman and Rubin, 1992). Each parameter has the $\hat{R}$ statistic associated with it (Gelman and Rubin, 1992), in the recent version (Vehtari et al., 2021); this is essentially the ratio of between-chain variance to within-chain variance (analogous to ANOVA). The $\hat{R}$ statistic should be approximately 1 ± 0.1 if the chain has converged.

To compare Bayesian models, different indicators can be adopted (Gelfand and Dey, 1994; Wasserman, 2000; Gelman et al., 2014). The sum of squared errors is a reasonable measure proposed. Although log-likelihood plays an important role in statistical model comparison, it also has some drawbacks, for example, the dependence on the number of parameters and on the sample size. A reasonable alternative is to evaluate a model through the log predictive density and its accuracy. Log pointwise predictive density (*lppd*) for a single value *y*_*i*_ is defined as Vehtari et al. (2017);


$$\begin{array}{l} logp\left(y_{i}|y\right)=log\int p\left(y_{i}|\theta \right)p\left(\theta |y\right)d\theta \end{array}$$

The log pointwise predictive density (*lppd*) is defined as the sum and can be computed using results from the posterior simulation


$$\begin{array}{l} lppd=\overset{n}{\underset{i=1}{\sum }}logp\left(y_{i}|y\right)=\overset{n}{\underset{i=1}{\sum }}log\int p\left(y_{i}|\theta \right)p\left(\theta |y\right)d\theta \end{array}$$

## 3. Fitting hierarchical bayesian models to the experimental data

Studies from our research group shed light on the interplay between slip motion and high-frequency vibrations (masking vibration) in the discrimination of velocity by touch (Dallmann et al., 2015; Picconi et al., 2022; Ryan et al., 2022). These and similar results are discussed in our recent review (Ryan et al., 2021). Using Bayesian hierarchical models, we combined two of these studies and evaluated the coherence of our findings across experiments. The two studies are summarized in Sections 3.1 and 3.2, respectively. Examples of the R and JAGS files for fitting our data are available in the following Github repository https://github.com/moskante/bayesian_models_psychophysics.

### 3.1. First data-set: The role of vibration in tactile speed perception

The data-set *touch-vibrations* was first published by Dallmann et al. (2015) and it is provided within the CRAN package *MixedPsy*. It consists of the forced-choice responses (i.e., the comparison stimulus is “faster” or “slower” than a reference) collected in a psychophysical study from nine human observers and the corresponding predictor variables. The task is as follows: In two separate intervals, participants were requested to compare the motion speed of a moving surface by touching it and reported whether it moved faster in the reference or the comparison stimulus. The speed of the comparison stimulus was chosen among seven values of speed ranging between 1.0 and 16.0 cm/s. In two separate blocks, participants performed the task either with masking vibrations (sinusoidal wave signal at 32 Hz) or without (control condition). Each speed and vibration combination was repeated 40 times in randomized order, resulting in a total of 560 trials for each participant.

According to Dallmann et al. (2015), GLMM with a probit link function was fitted to the data and the results presented in Supplementary Tables S1, S2 were obtained. Next, the data were fitted with a hierarchical Bayesian model in JAGS. Let $Y_{ij}^{h}$ indicates the number of “faster” responses for subject *i* at speed *x*_*j*_. Superscript *h* indicates the presence or absence of masking vibrations. That is, *h* = 0 masking vibrations were not present while *h* = 1 masking vibrations were present. $n_{ij}^{h}$ is the total number of trials for subject *i*, speed *x*_*j*_ and vibration condition *h*. The model is the following:


(9) $$\begin{array}{lll} Y_{ij}^{h} & \sim & Binom\left(\pi_{ij}^{h},n_{i,j}^{h}\right) \end{array}$$

(10) $$\begin{array}{lll} {\text{Φ}}^{-1}\left(\pi_{ij}^{h}\right) & = & \alpha_{i}^{h}+\beta_{i}^{h}x_{j}\;h=0,1 \end{array}$$

The following set of priors are assumed:


(11) $$\begin{array}{lll} \alpha_{i}^{h} & \sim & Norm\left(a^{h},\tau_{\alpha }^{h}\right) \end{array}$$

(12) $$\begin{array}{lll} \beta_{i}^{h} & \sim & Norm\left(b^{h},\tau_{\beta }^{h}\right) \end{array}$$

(13) $$\begin{array}{lll} \tau_{\alpha }^{h} & \sim & Gamma\left(1,0.001\right) \end{array}$$

(14) $$\begin{array}{lll} \tau_{\beta }^{h} & \sim & Gamma\left(1,0.001\right) \end{array}$$

(15) $$\begin{array}{lll} a^{h} & \sim & Norm\left(0,\sigma_{a}\right) \end{array}$$

(16) $$\begin{array}{lll} b^{h} & \sim & Norm\left(0,\sigma_{b}\right) \end{array}$$

(17) $$\begin{array}{lll} \sigma_{a} & \sim & Gamma\left(1,0.01\right) \end{array}$$

(18) $$\begin{array}{lll} \sigma_{b} & \sim & Gamma\left(1,0.01\right) \end{array}$$

The model in Equation (10) can be parameterized as follows to allow focus on parameter PSE and the slope $\beta_{i}^{h}$:


(19) $$\begin{array}{lll} Y_{ij}^{h} & \sim & Binom\left(\pi_{ij}^{h},n_{i,j}^{h}\right)\;h=0,1 \end{array}$$

(20) $$\begin{array}{lll} {\text{Φ}}^{-1}\left(\pi_{ij}^{h}\right) & = & -pse_{i}^{h}*\beta_{i}^{h}+\beta_{i}^{h}x_{j} \end{array}$$

(21) $$\begin{array}{lll} pse_{i}^{h} & \sim & Norm\left(PSE^{h},\tau_{PSE}^{h}\right) \end{array}$$

(22) $$\begin{array}{lll} \beta_{i}^{h} & \sim & Norm\left(b^{h},\tau_{b}^{h}\right) \end{array}$$

(23) $$\begin{array}{lll} \tau_{PSE}^{h} & \sim & Gamma\left(1,0.001\right) \end{array}$$

(24) $$\begin{array}{lll} \tau_{b}^{h} & \sim & Gamma\left(1,0.001\right) \end{array}$$

(25) $$\begin{array}{lll} PSE^{h} & \sim & Norm\left(0,\sigma_{PSE}\right) \end{array}$$

(26) $$\begin{array}{lll} b^{h} & \sim & Norm\left(0,\sigma_{b}\right) \end{array}$$

(27) $$\begin{array}{lll} \sigma_{PSE} & \sim & Gamma\left(1,0.01\right) \end{array}$$

(28) $$\begin{array}{lll} \sigma_{b} & \sim & Gamma\left(1,0.01\right) \end{array}$$

We used the Greek letter $\beta_{i}^{h}$ and the Latin letter *b*^*h*^ for the slope of subject *i* and the conditional value of slope common to all subjects, respectively. Similarly, we used the term $pse_{i}^{h}$ and *PSE*^*h*^ for the estimate of the PSE in subject *i* and the conditional estimate.

**Figure 1:**
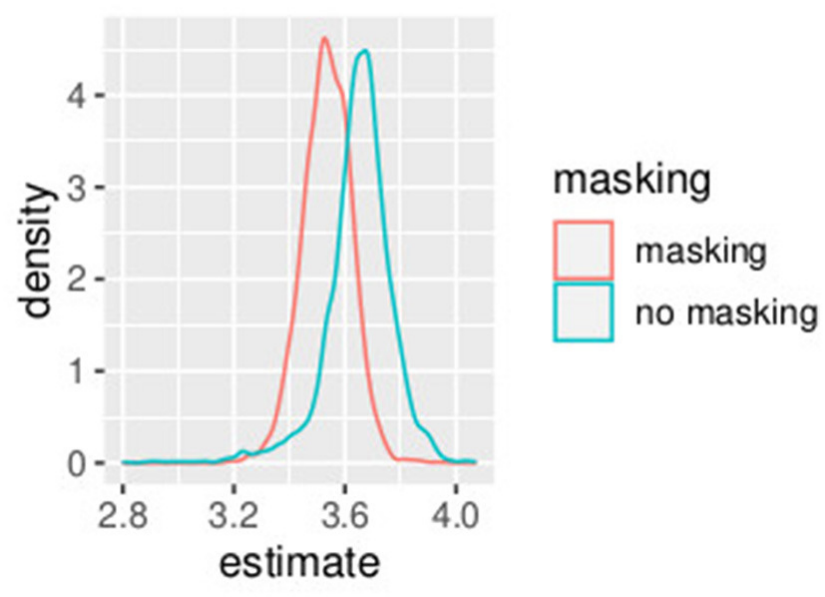
Posterior estimates of parameters *b*^*h*^ (slope). Experiment in Section 3.1.

**Figure 2:**
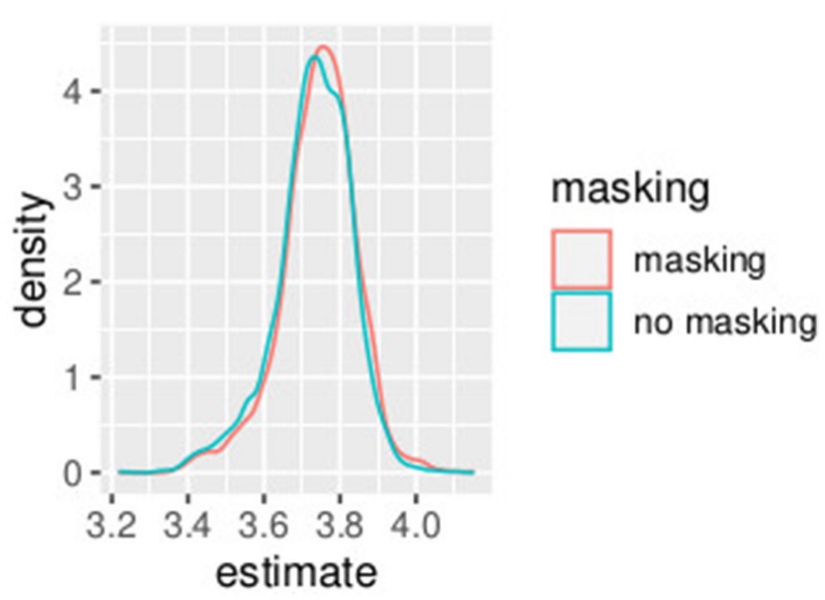
Posterior estimates of parameters *PSE*^*h*^. Experiment in Section 3.1.

In this first example, non-informative prior distributions were adopted and the hierarchical Bayesian model confirmed the results obtained with the GLMM, as expected. Supplementary Table S3 presents the posterior estimates of *a*^*h*^ and *b*^*h*^ as defined in Equations (10)–(19), while Supplementary Table S4 presents posterior estimates of *PSE*^*h*^ as defined (Equations 20–29). Comparing Supplementary Table S2 (GLMM) and Supplementary Table S4 (Bayesian model), the PSE estimates result very close and the uncertainty is very similar with the two model approaches. Figures 1, 2 show the posterior distribution of the two parameters of the model *b*^*h*^ and *PSE*^*h*^ as defined in Equations (22), (23) that are common to all the subjects. The slope of the model is slightly higher without masking vibrations (*b*^0^, in blue in the figure) as compared to masking vibrations (*b*^1^, in red in the figure). The difference in PSE is negligible.

We considered the overlap between the posterior distributions as a measure of similarities and differences between parameters, where overlapping is defined as the area intersected by the two distributions. Overlapping was computed as the proportion of the areas of the histograms belonging to the region shared by the two distributions. The idea of overlapping as a measure of similarity among data-sets or clusters is frequently used in different fields (Pastore and Calcagǹı, 2019; Mezzetti et al., 2022).

An effect of vibration is present for the intercept. The overlap between the distribution of *b*^0^ and *b*^1^, the slope of the model, is 0.04. The overlap of the posterior distributions of PSE, in presence of vibration versus absence of vibration, is 0.58. This is consistent with our GLMM analysis where we found a small (yet significant) difference in slope but no differences in PSE.

**Figure 3:**
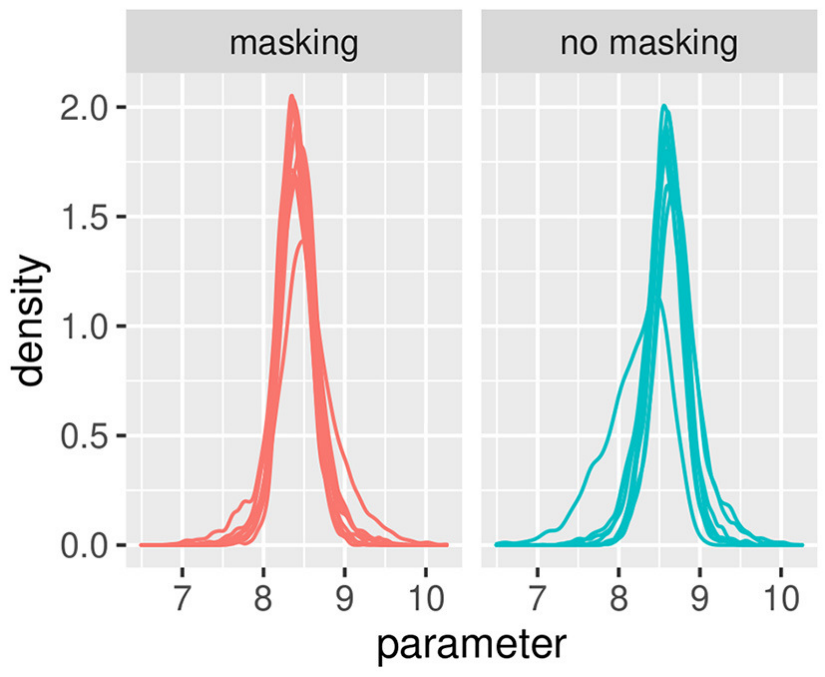
Posterior estimates of individual parameters of $pse_{i}^{h}$. The **(left)** figure illustrated with red lines represents conditions with masking vibrations, while the **(right)** figure illustrated with blue lines represents conditions without masking vibrations. Experiment in Section 3.1.

**Figure 4:**
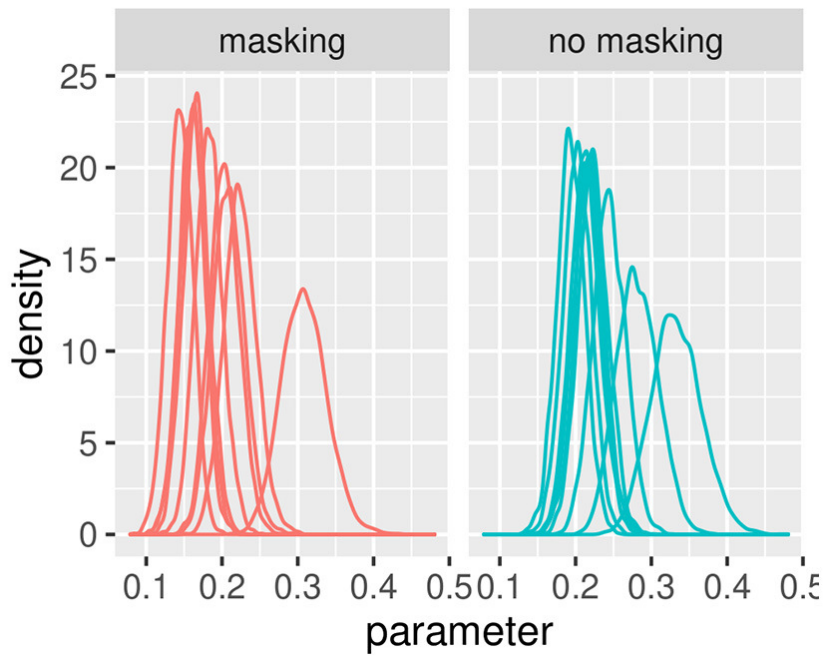
Posterior estimates of individual parameters of $\beta_{i}^{h}$. The **(left)** figure illustrated with red lines represents conditions with masking vibrations while the **(right)** figure illustrated with blue lines represents conditions without masking vibrations. Experiment in Section 3.1.

**Figure 5:**
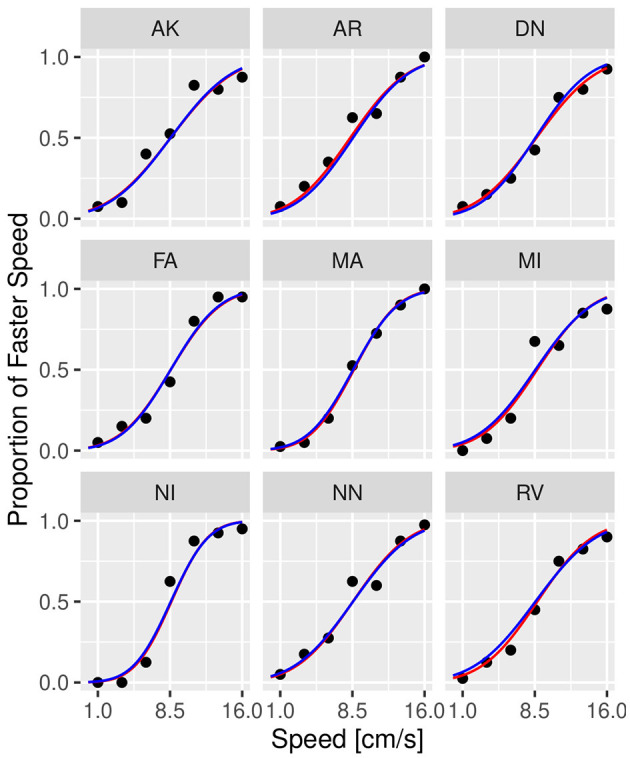
Psychometric functions of individual participants from Experiment 1 in conditions without masking vibrations. The scatter plot shows the observed (dots) versus predicted responses (solid lines) with data from individual participants illustrated in each panel. Blue lines correspond to the prediction by GLMM, while red lines correspond to predictions by the Bayesian model. Experiment in Section 3.1.

**Figure 6:**
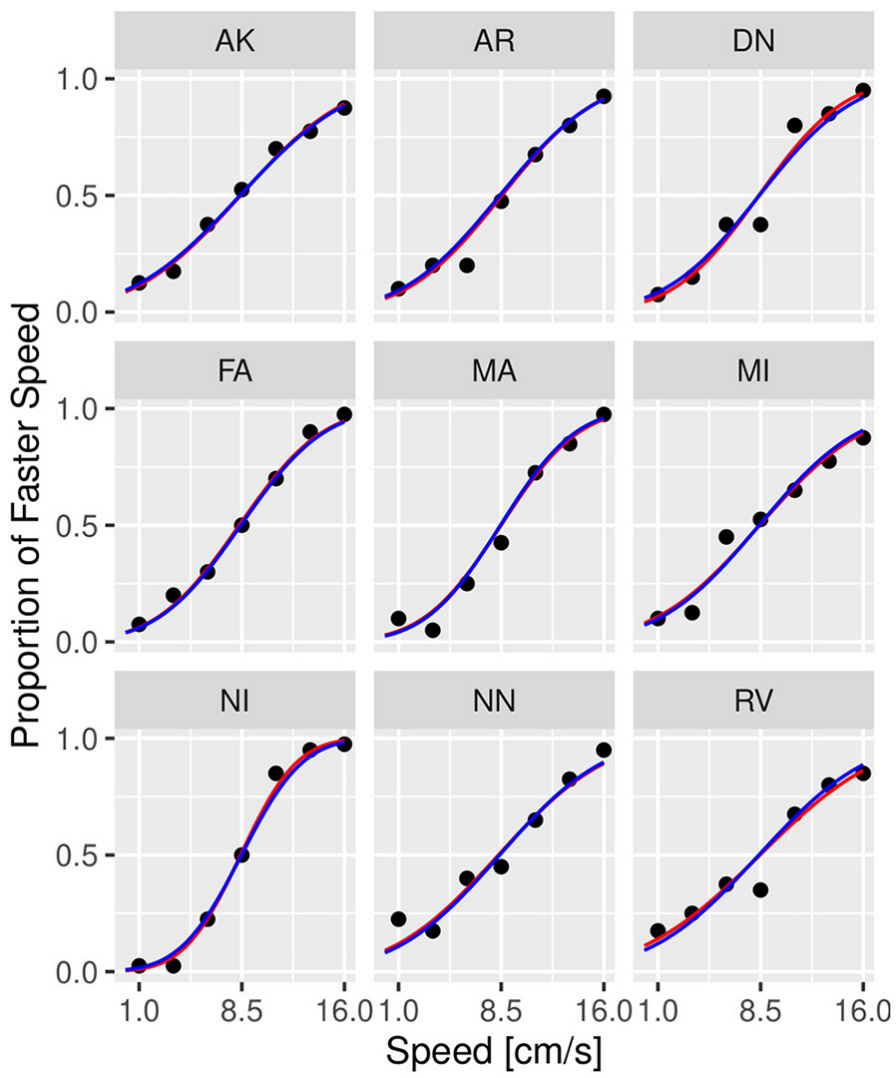
Psychometric functions of individual participants from Experiment 1 in conditions with 32 Hz masking vibrations. The scatter plot shows the observed (dots) versus predicted responses (solid lines) with data from individual participants illustrated in each panel. Blue lines correspond to the prediction by GLMM, while red lines correspond to predictions by the Bayesian model. Experiment in Section 3.1.

Figures 3, 4 illustrate the posterior distributions of the parameters of the individual psychometric function, as specified in Equations (11), (22). It is interesting to notice that between-subject variability is present for the slope (parameter $\beta_{i}^{h}$), while subjects show similar behavior in posterior distribution respect to PSE (parameter $pse_{i}^{h}$). In fact in Figure 3, the between individual variability of PSE is quite negligible. Finally, Figures 5, 6 compare the predictions of the GLMM and of the hierarchical Bayesian model across the nine participants. The predictions of the two models are almost identical. To conclude, since we used a non-informative prior, the outcome of the Bayesian model does not differ substantially from the GLMM that was used in the original study.

Different specifications of the prior distributions in Equations (24), (25) and in Equations (28), (29) were considered, based on the sum of squared errors and the uncertainties of parameters, measured with the length of credible intervals. In particular, alternative specification of Equations (22)–(25) was considered:


(29) $$\begin{array}{l} pse_{i}^{h}\sim Norm(PSE^{h},\tau_{PSE}^{i}) \end{array}$$

(30) $$\begin{array}{l} \beta_{i}^{h}\sim Norm\left(b^{h},\tau_{b}^{i}\right) \end{array}$$

(31) $$\begin{array}{l} \tau_{PSE}^{i}\sim Gamma\left(1,0.001\right) \end{array}$$

(32) $$\begin{array}{l} \tau_{b}^{i}\sim Gamma\left(1,0.001\right) \end{array}$$

Specifically, in the model earlier, each subject can have a different precision in the two parameters of PSE and slope—i.e., $\tau_{PSE}^{i}$ and $\tau_{b}^{i}$ may have different values depending on the participant. The previous choice of prior distributions assumed higher variability between subjects and evidenced a different outcome in the subject *NI* as compared to the others with respect to the intercept and the slope. The alternative specifications of prior distributions in Equations (30)–(33) provide similar values with respect to the sum of squared errors, and the length of credible intervals for the PSE was slightly lower than the model in Equations (28), (29). Table 1 shows the frequentist approach (GLMM) and the different specifications of the Bayesian model. Comparing the models with respect to the uncertainties in PSE estimation and model fitting, we justify the choice of the model proposed.

**Table 1:** Comparison between the different models in data-set *touch-vibrations*.

**Model**	**Effects**	**Log likelihood**	**LPPD**	**Sum errors**	**95*%* CI of PSE**	**Width CI**
GLMM	Individual	-	-	-		
	Overall	–284.42	-	0.62	(0.52, 0.59)	0.07
Bayesian 1	Individual	–276.23	–14231.6 (1081.1)	0.42		
	Overall			0.61	(0.49, 0.55)	0.06
Bayesian 2	Individual	–278.03	–14323.0 (3.6)	0.41		
	Overall			0.63	(0.49, 0.50)	0.01
Bayesian 3	Individual	–276.29	14163.2 (2.0)	0.40		
	Overall			0.61	(0.57, 0.61)	0.04
Bayesian 4	Individual	–275.86	-14155.8 (2.3)	0.39		
	Overall			0.61	(0.58, 0.63)	0.05

For each model, we showed the log-likelihood and the LPPD of the model, and the sum or squared errors and length of the Credible Intervals of the PSE. The first two lines refer to the GLMM as described by Dallmann et al. (2015). The third and the fourth lines show the Bayesian model 1, as specified in Equations (20)–(29). In the fifth and the sixth lines, the Bayesian model 2 is shown, with a different specification of the prior distributions as in (30)–(33). In the Bayesian model 3, the distribution of $\tau_{\alpha ,\beta }^{h}\sim Gamma\left(1,0.1\right)$ was used, as in (22)–(25). The last two lines show the Bayesian model 4 with the distribution of τ is $\tau_{\alpha ,\beta }^{i}\sim Gamma\left(1,0.1\right)$. This means that the variability of the PSE and the slope was allowed to be different for each participant.

### 3.2. Second data-set: Tactile speed discrimination in people with type 1 diabetes

The second data-set, *touch-diabetes*, includes data from 60 human participants that were tested in a speed discrimination task similar to the one described in Section 3.1. The experimental procedure and the results are detailed by Picconi et al. (2022). Participants were divided into three groups, with 20 participants per group: healthy controls, participants with diabetes with mild tactile dysfunction, and participants with diabetes with moderate tactile dysfunction. The three groups were labeled as *controls, mild, and moderate*, respectively. As in *touch-vibration*, this experiment consisted of a force-choice, speed discrimination task. In each of the 120 trials, participants were requested to indicate whether a contact surface moved faster during a comparison or a reference stimulus interval. For this experiment, a smooth surface consisting of a glass plate was used. The motion speed of the comparison stimuli were as chosen pseudo-randomly from a set of five values ranging from 0.6 to 6.4 cm/s, with the speed of the reference stimulus equal to 3.4 cm/s. Participants performed the task with and without masking vibrations, with masking stimuli consisting of sinusoidal vibrations at 100 Hz.

As in the original study, we used the GLMM in Equations (34)–(36) to fit the data across groups and across masking vibration conditions:


(33) $$\begin{array}{l} Y_{ij}^{h}\sim Binom\left(\pi_{ij}^{h},n_{i,j}^{h}\right)\;h=0,1 \end{array}$$

(34) $$\begin{array}{l} \Phi^{-1}\left(\pi_{ij}^{h}\right)=\alpha_{i}^{h}+\beta_{i}^{h}x_{j} \end{array}$$

The response variable $Y_{ij}^{h}$ is the number of “faster” responses for subject *i* at speed *x*_*j*_. The suprascript *h* = 0 represents conditions without masking vibrations and *h* = 1 represents conditions with masking vibration. The variable $n_{ij}^{h}$ is the total number of trials. Considering two dummy variables for the two groups of participants with diabetes, *mild* (indicated with subscript 2) and *moderate* (indicated with subscript 3) patients with diabetes, the individual model with fixed effects is rewritten as:


(35) $$\begin{array}{l} \Phi^{-1}\left(\pi_{ij}^{h}\right)=\alpha^{h}+\alpha_{2}^{h}+\alpha_{3}^{h}+\beta^{h}x_{j}+\beta_{2}^{h}x_{j}+\beta_{3}^{h}x_{j} \end{array}$$

We used the packages MixedPsy (Balestrucci et al., 2022) and lme4 (Bates et al., 2015) for model fitting. Supplementary Tables S5, S6 report results for the frequentist approach (GLMM). The slope of the model (referred to as *tactile sensitivity* in the study) was different across the three groups, with controls performing significantly better in the task than people with mild and moderate tactile dysfunctions. The difference between groups was larger without masking vibrations. As in the first data-set, masking vibrations reduced the values of the slope across all groups. We computed the values of PSE for all groups and conditions, see Supplementary Table S6. We expected no significant change in PSE, both between masking vibration conditions and between groups. This is because, in this task, the cues and the sensory noise are the same in the reference and comparison stimulus.

As in the previous example, we re-analyzed the data with a Bayesian hierarchical model. Let *i* indicates subject, *j* speed, *h* masking or no masking, and *k* indicates group.

Similar to the analysis of the first data-set, the model was parameterized with respect to the PSE and the slope:


(36) $$\begin{array}{l} Y_{ij}^{h}\sim Binom\left(\pi_{ij}^{h},n_{i,j}^{h}\right) \end{array}$$

(37) $$\begin{array}{l} \Phi^{-1}\left(\pi_{ij}^{h}\right)=-pse_{i}^{h}*\beta_{i}^{h}+\beta_{i}^{h}x_{j}\;h=0,1 \end{array}$$

The following prior and hyper-prior distributions are assumed:


(38) $$\begin{array}{l} pse_{i}^{h}\sim Norm\left(PSE_{k}^{h},\tau_{PSE,k}^{h}\right)\;h=0,1k=1,2,3 \end{array}$$

(39) $$\begin{array}{l} \beta_{i}^{h}\sim Norm\left(b_{k}^{h},\tau_{\beta ,k}^{h}\right) \end{array}$$

(40) $$\begin{array}{l} \tau_{PSE,k}^{h}\sim Gamma\left(1,0.001\right) \end{array}$$

(41) $$\begin{array}{l} \tau_{\beta ,k}^{h}\sim Gamma\left(1,0.001\right) \end{array}$$

(42) $$\begin{array}{l} PSE_{k}^{h}\sim Norm\left(0,\sigma_{PSE}\right) \end{array}$$

(43) $$\begin{array}{l} b_{k}^{h}\sim Norm\left(0,\sigma_{b}\right) \end{array}$$

(44) $$\begin{array}{l} \sigma_{PSE}\sim Gamma\left(1,0.01\right) \end{array}$$

(45) $$\begin{array}{l} \sigma_{b}\sim Gamma\left(1,0.01\right) \end{array}$$

**Figure 7:**
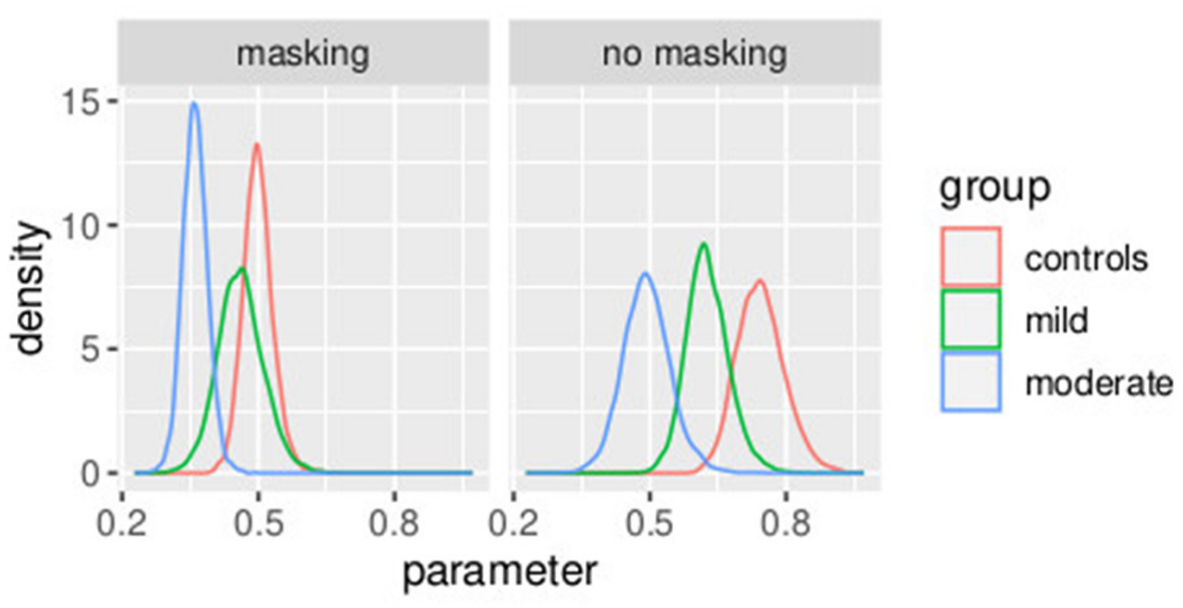
Posterior distributions of parameters $b_{k}^{h}$ from the second stage of the hierarchical model. Experiment in Section 3.2.

The mean and the credible intervals of the parameters of the models $b_{k}^{h}$ (slope) and $PSE_{k}^{h}$, as defined in Equations (34)–(46), are reported in Supplementary Table S7. The results confirmed the difference in slopes between the groups and between conditions. In conditions without masking vibrations, the slope was the highest in controls followed by the mild and moderate groups. The mean of the slope in controls is higher than the credible intervals of the mild group. Similarly, the mean of the slope of the mild group is higher than the credible intervals of the moderate group. The same effect can be observed in the masking vibration conditions, although the difference in slope is smaller between the control and mild groups. In Figure 7, the posterior distributions of the slope of the model are shown. We can observe the two effects of group (ordered from controls to moderate) and masking conditions. In particular, the group with moderate tactile dysfunction (illustrated in blue) is the one with the lowest values of slope.

**Figure 8:**
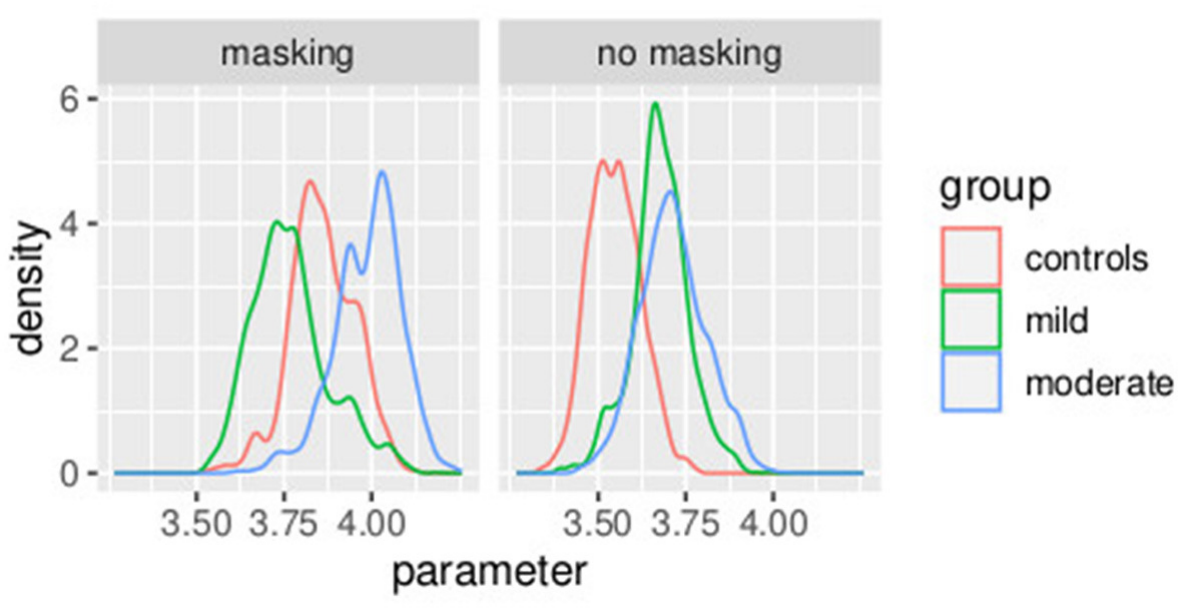
Posterior distributions of parameters of the second stage of the hierarchical model $\text{PS}{\text{E}}_{k}^{h}$. Experiment in Section 3.2.

**Figure 9:**
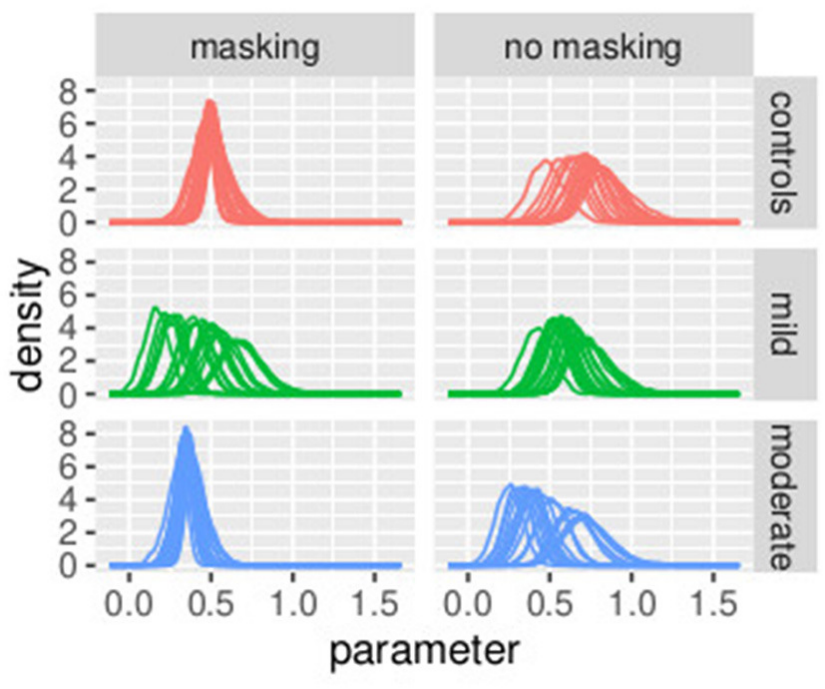
Posterior distributions of parameters of the first stage of the hierarchical model $\beta_{i}^{h}$, by group and masking condition. Experiment in Section 3.2.

**Figure 10:**
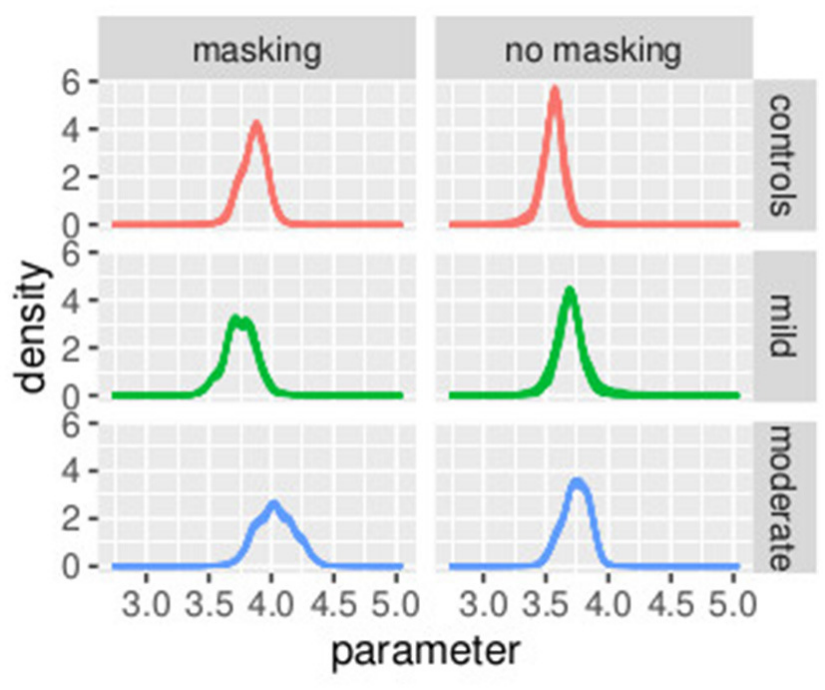
Posterior distributions of parameters of the first stage of the hierarchical model $PSE_{i}^{h}$, by group and masking condition. Experiment in Section 3.2.

In Figure 8, the posterior distributions of the PSE values, as specified in Equations (37)–(46) are shown. Uncertainties in the parameters $PSE_{k}^{h}$ were comparable between the frequentist and the Bayesian models. This was expected because in this Bayesian model, we used a non-informative prior. Masking vibrations had a large effect on the slope and a much smaller effect on the PSE. Within the control group, the overlap between the posterior distributions of PSE with masking versus no masking is 0.04, and the overlap between the posterior distribution of the slope between masking and no masking is <0.01. This supports our finding that masking vibration reduced tactile sensitivity. In Figures 9, 10, the posterior distributions of the individual parameters β_*i*_ and *pse*_*i*_ are shown. Again, it is interesting to notice that the posterior estimates of PSE have low subject variability. The individual posterior distributions show a higher overlapping, refer to Figure 10 for an almost perfect overlapping. Within groups, variability is lower for PSE compared to posterior distributions of the parameters representing the slopes.

## 4. Combined analysis of the two experiments

In this section, we propose two different approaches for the joint analysis of the two studies. In Section 4.1, the prior distributions of the parameters relative to the second study are defined from the data of the first study. In Section 4.2, a model approach based on the power prior distribution explained in Section 2.1 was applied to combine the two data-sets *touch-vibrations* and *touch-diabetes*.

The data-set *touch-vibrations* is considered historical data and indicated a *D*_0_ = (*n*_0_, *y*_0_, *x*_0_), where *n*_0_ is the sample size of the historical data, *y*_0_ is the number of “faster” responses the *n*_0_ × 1 response vector, in this case number of, *x*_0_ is a *n*_0_ × 1 vector of speed. The data-set *touch-diabetes* indicated the current study, we restrict the analysis only to the control group, we discarded the two diabetic groups because of their reduced tactile sensitivity. Data are denoted by *D* = (*n, y, x*), where *n* denotes the sample size, *y* denotes the *n* × 1 response vector, the number of “faster” responses, and *x* the *n* × 2 matrix of covariates, indicator of cluster and speed.

### 4.1. Prior distribution defined on the first experiment

The two data-sets are jointly analyzed. Equations (34)–(46) are rewritten incorporating model (Equations 10, 11) in order to combine the two studies as follows:


(46) $$\begin{array}{l} Y_{ij}^{h}\sim Binom\left(\pi_{ij}^{h},n_{i,j}^{h}\right) \end{array}$$

(47) $$\begin{array}{l} \Phi^{-1}\left(\pi_{ij}^{h}\right)=\alpha_{i}^{h}+\beta_{i}^{h}x_{j} \end{array}$$

(48) $$\begin{array}{l} Y_{0ij}^{h}\sim Binom\left(\pi_{0ij}^{h},n_{0i,j}^{h}\right) \end{array}$$

(49) $$\begin{array}{l} \Phi^{-1}\left(\pi_{0ij}^{h}\right)=\alpha_{0i}^{h}+\beta_{0i}^{h}x_{0j} \end{array}$$

Because of Weber's Law, the sensitivity to speed and, therefore, the slope depends on the value of the stimulus. To address this issue, to combine the two experiments, we used the conversion factor in Equation (55).


(50) $$\begin{array}{l} \alpha_{i}^{h}\sim Norm\left(a^{h},\tau_{a}^{h}\right) \end{array}$$

(51) $$\begin{array}{l} \beta_{i}^{h}\sim Norm\left(b^{h},\tau_{b}^{h}\right) \end{array}$$

(52) $$\begin{array}{l} \alpha_{0i}^{h}\sim Norm\left(a_{0}^{h},\tau_{a0}^{h}\right) \end{array}$$

(53) $$\begin{array}{l} \beta_{0i}^{h}\sim Norm\left(b_{0}^{h},\tau_{b0}^{h}\right) \end{array}$$

(54) $$\begin{array}{l} a^{h}\sim Norm\left(a_{0}^{h},\sigma_{a}^{h}\right) \end{array}$$

(55) $$\begin{array}{l} b^{h}\sim Norm\left(b_{0}^{h}\times \frac{\bar{x}}{\bar{x_{0}}},\sigma_{b}^{h}\right) \end{array}$$

(56) $$\begin{array}{l} a_{0}^{h}\sim Norm\left(0,\sigma_{a}^{0}\right) \end{array}$$

(57) $$\begin{array}{l} b_{0}^{h}\sim Norm\left(0,\sigma_{b}^{0}\right) \end{array}$$

(58) $$\begin{array}{l} \sigma_{k}^{h}\sim Gamma\left(1,0.01\right)\;h=0,1,2\;k=a,b \end{array}$$

(59) $$\begin{array}{l} \tau_{k}^{h}\sim Gamma\left(1,0.01\right)\;h=1,2\;k=a0,a,b0,b \end{array}$$

From the posterior estimates of parameters $\sigma_{a}^{h}$ and $\sigma_{b}^{h}$, we can gain information about whether the combination of two studies is appropriate for the same model. The posterior distributions of the precision parameters indicate a good agreement between the two studies and confirm the suitability of the choice for the prior distribution. High-posterior estimates of the precision of the prior distribution indicate good agreement between prior distribution and data.

### 4.2. Power prior model

Recalling Section 2.1, the prior distribution of parameters θ = (α, β) is defined as follows:


(60) $$\begin{array}{l} \pi \left(\theta |D_{0},a_{0}\right)\propto L{\left(\theta |D_{0}\right)}^{a_{0}}\pi_{0}\left(\theta \right). \end{array}$$

The power parameter *a*_0_ represents the weight of the historical data relative to the likelihood of the current study. The parameters represent how much data from the previous study is to be used in the current study. There are two special cases for *a*_0_, the first case *a*_0_ = 0 results in no incorporation of the data from the previous study relative to the current study. The second case *a*_0_ = 1 results in full incorporation of the data from the previous study relative to the current study. Therefore, *a*_0_ controls the influence of the data gathered from previous studies that is similar to the current study. This control is important when the sample size of the current data is quite different from the sample size of historical data or where there is heterogeneity between two studies (Ibrahim and Chen, 2000).

In Table 2, a comparison between all the models obtained by varying the parameter *a*_0_ is shown. The choice of the value for *a*_0_ is implemented by model comparison, taking into account the log-likelihood, the log point-wise predictive density, the sum of squared errors, of both the level of the model, that are the individual and overall model. Moreover, a comparison of the uncertainty in PSE estimation is computed. The uncertainty decreases as *a*_0_ increases indicating that we are updating our informative knowledge for the correct model use. The likelihood increases as the value of *a*_0_ increases. The measures of goodness of fit of the models are very similar increasing the value of *a*_0_. We decide to favor the model that lowers the uncertainties in the estimation, that is the model with *a*_0_ = 0.7.

**Table 2:** Comparison between the different models obtained by varying values of *a*_0_ in Equation (60), as illustrated in subsection 4.2.

***a*_0_**	**Effects**	**Log likelihood**	**LPPD**	**Sum errors**	**CI of PSE**	**Width CI**
0	Individual	–291.76	–3169.67 (9.91)	2.24		
	Overall			2.68	(0.32, 0.37)	0.05
0.1	Individual	–292.85	–3183.66 (8.66)	2.24		
	Overall			2.64	(0.27, 0.26)	0.01
0.2	Individual	–293.47	–3202.41 (7.18)	2.21		
	Overall			2.64	(0.24, 0.27)	0.03
0.3	Individual	–294.07	–3221.91 (8.45)	2.21		
	Overall			2.64	(0.22, 0.27)	0.05
0.4	Individual	–295.08	–3243.93 (7.02)	2.20		
	Overall			2.65	(0.25, 0.24)	0.01
0.5	Individual	-295.48	-3242.41 (8.75)	2.20		
	Overall			2.66	(0.21, 0.25)	0.04
0.6	Individual	-296.00	–3253.47 (13.02)	2.20		
	Overall			2.66	(0.19, 0.27)	0.08
0.7	Individual	–296.02	–3256.63 (8.33)	2.23		
	Overall			2.68	(0.18, 0.22)	0.04
0.8	Individual	–296.84	–3276.64 (7.70)	2.1		
	Overall			2.68	(0.18, 0.25)	0.07
0.9	Individual	-296.77	–3268.68 (12.03)	2.23		
	Overall			2.68	(0.18, 0.2)	0.02
1	individual	–297.27	–3268.46 (10.7)	2.22		
	Overall			2.7	(0.17, 0.24)	0.07

For each model, we showed the log-likelihood, the LPPD of the model, the sum or squared errors, and length of the Credible Intervals of the PSE.

**Figure 11:**
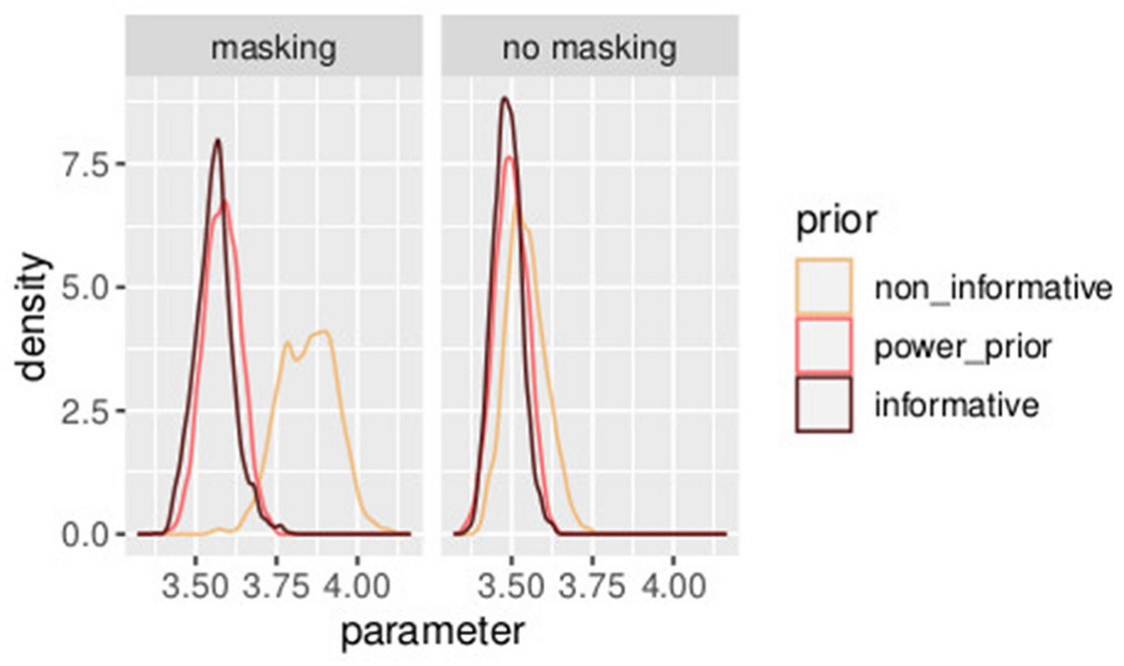
Posterior distributions of parameters *PSE*^*h*^ with different prior distributions for different values of *a*_0_. The model with the informative prior (*a*_0_ = 1.0) is illustrated in dark brown, the one with the power prior (α_0_ = 0.7) in orange, and the one with the non-informative prior (α_0_ = 0.0) in yellow. Experiment in Section 3.2.

**Figure 12:**
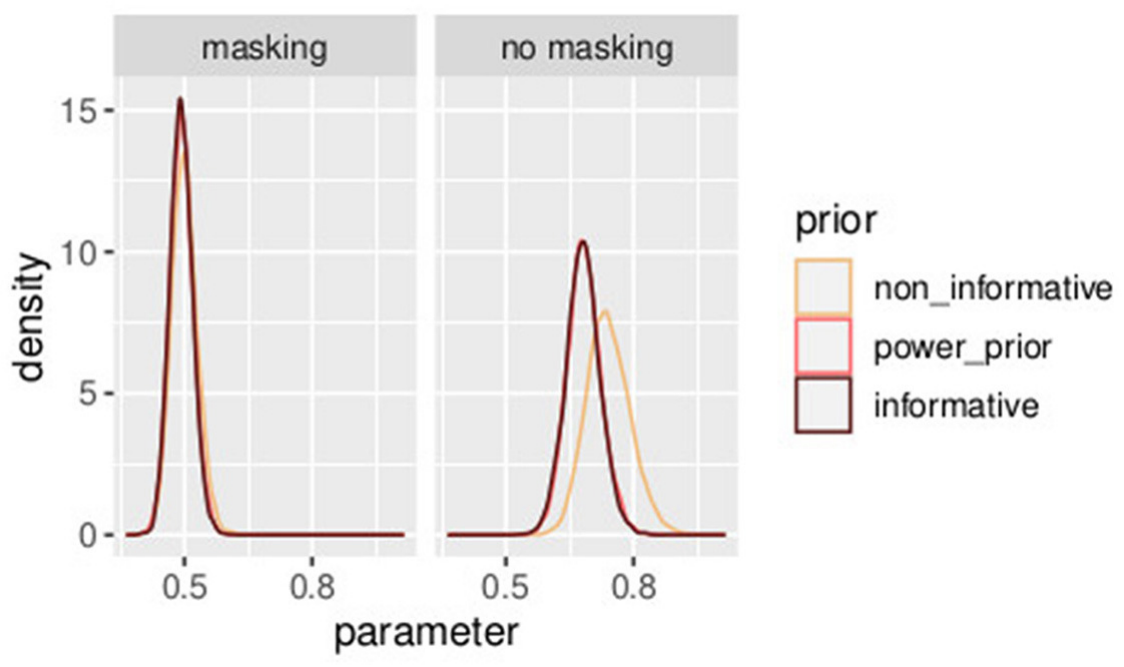
Posterior distributions of parameters *b*^*h*^ with different prior distributions for different values of *a*_0_. The model with the informative prior (*a*_0_ = 1.0) is illustrated in dark brown, the one with the power prior (*a*_0_ = 0.7) in orange, and the one with the non-informative prior (*a*_0_ = 0.0) in yellow. Experiment in Section 3.2.

In Table 3, three different prior distributions are compared. On one hand, an informative prior is assumed following Section 3.2; on the other hand, the first experiment is used to improve the understanding of experiment 2. A combination of the two studies [as in Equations (47)–(59)] illustrated in Section 4.1 is compared with power prior as in Section (4.2). In Figures 11, 12, a comparison of the posterior distributions of *PSE* and β, in the control group, obtained according to the three different prior distributions is shown. Again we favor the model that lowers the uncertainties of posterior estimates. Overall, combining the two studies with the power prior approach reduced the posterior estimate of the model parameters as can be clearly seen by comparing the three distributions in the figures.

**Table 3:** Comparison between the different priors assumed for the data-set *touch-diabetes*.

**Model**	**Effects**	**Log like**	**LPPD**	**Sum errors**	**CI of PSE**	**Width CI**
Non Informative 1	Individual	–285.92	−3026.4(3.4)	2.04		
	Overall			2.68	(0.3, 0.35)	0.05
Non Informative 2	Individual	–291.66	−3153.9(6.2)	2.25	
	Overall			2.67	(0.26, 0.32)	0.06
Non Informative 3	Individual	-283.74	−3000.3(2.8)	1.91	
	Overall			2.68	(0.31, 0.39)	0.08
Informative Prior Subsection 4.1	Individual	-292.14	−3156.2(7.8)	2.2	
	Overall			2.65	(0.25, 0.36)	0.11
Informative Prior Subsection 4.2 with *a*_0_ = 0.7	Individual	–296.02	−3256.63(8.33)	2.23		
	Overall			2.68	(0.18, 0.22)	0.04

For each model, we showed the log-likelihood and the LPPD of the model, and the Sum or Squared Errors and the Credible Intervals of the PSE. The first three models refer to non-informative prior as illustrated in Equations (37)–(46). The first three models differ for hyperparameters in the Gamma distribution in Equations (41), (42). Non Informative 1 assumes τ_β, *PSE*_~*Gamma*(1, 0.01). Non Informative 2 assumes τ_β, *PSE*_~*Gamma*(1, 0.001) and Non Informative 3 assumes τ_β, *PSE*_~*Gamma*(0.1, 0.01). The fourth and fifth models refers to the joint analyzes of the two data-sets. In particular, the fourth model refers to prior illustrated in Section 4.1. The fifth model refers to the prior illustrated in Section 4.2 with *a*_0_ equal to 0.7.

## 5. Conclusion

In this study, we compared the outcome of a Bayesian approach to a frequentist mixed model (GLMM) approach. The comparison showed the importance of incorporating informative prior knowledge from previous studies for data analysis.

We re-analyzed data from two studies using GLMM and Bayesian models. First, we applied GLMM and four different Bayesian models to the data-set described by Dallmann et al. (2015). We compared the log-likelihood, LPPD, the sum of errors between the different models, and confidence interval of the two parameters of slope and PSE. The Bayesian approach allowed for more flexibility in the model fitting (see Table 1). Next, we applied Bayesian models to the second data-set for re-analysis of the results described by Picconi et al. (2022). With a non-informative prior, the Bayesian approach confirmed the estimation of the parameters of the frequentist model. Finally, we ran a joint analysis of the two data-sets using two different approaches, either by using the first data-set to choose the parameters of the prior or by using the power prior method. The informative prior in the power prior method reduced the credible intervals of the PSE and justified the choice of the model, as shown in Tables 2, 3.

The Bayesian approach provides useful features for the in-depth analysis of psychophysical data. Through a Bayesian approach, the random effects are estimated parameters, like the fixed effects, with the advantage of obtaining credible intervals for both the quantities. This allowed to estimate the effect of individual participants and the reliability of each of them. For example, in Figure 4, it is possible to identify a single participant with increased variability and higher slope as compared to the rest of the group. Potentially, this will simplify the identification of outliers or sources of unobserved variability. Another advantage of the hierarchical Bayesian approach is the possibility to incorporate information from past studies to reduce the uncertainty of the estimate. For example, compare the width of the three distributions in Figures 11, 12, with the non-informative prior having the larger width, i.e., the higher variance. This will increase the power of the analysis. Finally, this approach allowed quantifying the coherence of multiple studies on a related topic through the parameter *a0*. The greater the value of *a0*, the higher the coherence across the studies.

Hierarchical modeling is a natural tool for combining several data-sets or incorporating prior information. In the current study, the method presented by Chen and Ibrahim (2006) has been used that provides a formal connection between the power prior and hierarchical models for the class of generalized linear models. Understanding the impact of priors on the current data and subsequently making decisions about these priors is fundamental for the interpretation of data (Koenig et al., 2021). One of the assumptions of the power prior approach is the existence of a common set of parameters for the old and current data and this assumption may not be met in practice. An alternative approach to incorporate historical data has been proposed by Neuenschwander et al. (2010) and van Rosmalen et al. (2018). This other method is based on meta-analytic techniques (MAP) and assumes exchangeability between old and current parameters.

Incorporating previous knowledge and insight into the estimation process is a promising tool (Van de Schoot et al., 2017) that is particularly relevant in studies with small sample sizes, as is often in psychophysical experiments. In our case, the sample size of the first data-set differed from the sample size of the second data-set. To take this into account, the power prior approach allowed us to assign a different weight to the historical data and the current data. It is possible to purposefully choose the hyperparameters of the prior, τ, to increase the precision of the posterior estimate. Zitzmann et al. (2015) suggested to specify a slightly informative prior to the group-level variance. As shown in Section 4, diffuse priors produce results that are aligned with the likelihood. On the other hand, using an informative prior that is relatively far from the likelihood, produces a shift in the posterior. It is possible to conduct a prior sensitivity analysis to fully understand its influence on posterior estimates (Van de Schoot et al., 2017).

Uncertainty quantification is an important issue in psychophysics. Hierarchical Bayesian models allow the researcher to estimate the uncertainty at a group level and the one specific to individual participants. This model approach will have an important impact on the evaluation of psychometric functions in psychophysical data.

## Data availability statement

Publicly available datasets were analyzed in this study. This data can be found at: https://github.com/moskante/bayesian_models_psychophysics.

## Author contributions

MM: conceptualization, methodology, visualization, software, formal analysis, writing—original draft, and writing—reviewing and editing. CR: conceptualization, data curation, visualization, software, writing—original draft preparation, and writing—review and editing. PB: conceptualization, software, and writing—reviewing and editing. FL: conceptualization, data curation, and writing—reviewing and editing. AM: conceptualization, data curation, visualization, software, formal analysis, writing—original draft, and writing—reviewing and editing. All authors contributed to the article and approved the submitted version.

## References

[B1] Agresti A. (2002). Categorical Data Analysis, Vol. 482. Hoboken, NJ: John Wiley & Sons.

[B2] Alcalá-Quintana R. García-Pérez M. A. (2004). The role of parametric assumptions in adaptive bayesian estimation. Psychol. Methods 9, 250. 10.1037/1082-989X.9.2.250 15137892

[B3] Balestrucci P. Ernst M. O. Moscatelli A. (2022). Psychophysics with R: the R Package MixedPsy. bioRxiv 2022.06.20.496855. 10.1101/2022.06.20.496855

[B4] Bates D. Mächler M. Bolker B. Walker S. (2015). Fitting linear mixed-effects models using lme4. J. Stat. Softw. 67, 1–48. 10.18637/jss.v067.i01

[B5] Chen M.-H. Ibrahim J. G. (2006). The relationship between the power prior and hierarchical models. Bayesian Anal. 1, 551–574. 10.1214/06-BA118

[B6] Dal'Bello L. R. Izawa J. (2021). Task-relevant and task-irrelevant variability causally shape error-based motor learning. Neural Netw. 142, 583–596. 10.1016/j.neunet.2021.07.015 34352492

[B7] Dallmann C. J. Ernst M. O. Moscatelli A. (2015). The role of vibration in tactile speed perception. J. Neurophysiol. 114, 3131–3139. 10.1152/jn.00621.2015 26424580 PMC4686298

[B8] Eggleston B. S. Ibrahim J. G. Catellier D. (2017). Bayesian clinical trial design using markov models with applications to autoimmune disease. Contemp Clin. Trials 63, 73–83. 10.1016/j.cct.2017.02.004 28188841 PMC5787855

[B9] Fong Y. Rue H. Wakefield J. (2010). Bayesian inference for generalized linear mixed models. Biostatistics 11, 397–412. 10.1093/biostatistics/kxp053 19966070 PMC2883299

[B10] Foster D. H. Zychaluk K. (2009). Model-free estimation of the psychometric function. J. Vis. 9, 30–30. 10.1167/9.8.30 PMC2826188 19633355

[B11] Fox J.-P. Glas C. A. (2001). Bayesian estimation of a multilevel irt model using gibbs sampling. Psychometrika 66, 271–288. 10.1007/BF02294839

[B12] Gelfand A. E. Dey D. K. (1994). Bayesian model choice: asymptotics and exact calculations. J. R. Stat. Soc. B 56, 501–514. 10.1111/j.2517-6161.1994.tb01996.x

[B13] Gelman A. Carlin J. B. Stern H. S. Rubin D. B. (1995). Bayesian Data Analysis. New York, NY: Chapman and Hall; CRC.

[B14] Gelman A. Hwang J. Vehtari A. (2014). Understanding predictive information criteria for bayesian models. Stat. Comput. 24, 997–1016. 10.1007/s11222-013-9416-2

[B15] Gelman A. Rubin D. B. (1992). Inference from iterative simulation using multiple sequences. Stat. Sci. 7, 457–472. 10.1214/ss/1177011136

[B16] Houpt J. W. Bittner J. L. (2018). Analyzing thresholds and efficiency with hierarchical bayesian logistic regression. Vision Res. 148, 49–58. 10.1016/j.visres.2018.04.004 29678536

[B17] Ibrahim J. G. Chen M.-H. (2000). Power prior distributions for regression models. Stat. Sci. 15, 46–60. 10.1214/ss/1009212673

[B18] Ibrahim J. G. Chen M.-H. Gwon Y. Chen F. (2015). The power prior: theory and applications. Stat. Med. 34, 3724–3749. 10.1002/sim.6728 26346180 PMC4626399

[B19] Ibrahim J. G. Chen M.-H. MacEachern S. N. (1999). Bayesian variable selection for proportional hazards models. Can. J. Stat. 27, 701–717. 10.2307/3316126

[B20] Johnston A. Bruno A. Watanabe J. Quansah B. Patel N. Dakin S.. (2008). Visually-based temporal distortion in dyslexia. Vision Res. 48, 1852–1858. 10.1016/j.visres.2008.04.029 18589473

[B21] Knoblauch K. Maloney L. T. (2012). Modeling Psychophysical Data in R. New York, NY: Springer New York.

[B22] Koenig C. Depaoli S. Liu H. Van De Schoot R. (2021). Moving beyond non-informative prior distributions: achieving the full potential of bayesian methods for psychological research. Front. Psychol. 12, 809719. 10.3389/fpsyg.2021.809719 34956030 PMC8695424

[B23] Kruschke J. (2014). Doing Bayesian data analysis: A tutorial with R, JAGS, and Stan. Cambridge, MA: Academic Press.

[B24] Kuss M. Jäkel F. Wichmann F. A. (2005). Bayesian inference for psychometric functions. J. Vis. 5, 8–8. 10.1167/5.5.8 16097878

[B25] Linares D. López-Moliner J. (2016). quickpsy: an R package to fit psychometric functions for multiple groups. R J. 8, 122–131. 10.32614/RJ-2016-008

[B26] McElreath R. (2020). Statistical Rethinking: A Bayesian Course With Examples in R and Stan. New York, NY: Chapman and Hall; CRC.

[B27] Mezzetti M. Borzelli D. d'Avella A. (2022). A bayesian approach to model individual differences and to partition individuals: case studies in growth and learning curves. Stat. Methods Appl. 31, 1245–1271. 10.1007/s10260-022-00625-6

[B28] Morrone M. C. Ross J. Burr D. (2005). Saccadic eye movements cause compression of time as well as space. Nat. Neurosci. 8, 950–954. 10.1038/nn1488 15965472

[B29] Moscatelli A. Balestrucci P. (2017). Psychophysics with R: the R package MixedPsy. R package version 1.0(0).

[B30] Moscatelli A. Bianchi M. Serio A. Terekhov A. Hayward V. Ernst M. O.. (2016). The change in fingertip contact area as a novel proprioceptive cue. Curr. Biol. 26, 1159–1163. 10.1016/j.cub.2016.02.052 27068417 PMC4865678

[B31] Moscatelli A. Mezzetti M. Lacquaniti F. (2012). Modeling psychophysical data at the population-level: the generalized linear mixed model. J. Vis. 12, 26–26. 10.1167/12.11.26 23104819

[B32] Moscatelli A. Scotto C. R. Ernst M. O. (2019). Illusory changes in the perceived speed of motion derived from proprioception and touch. J. Neurophysiol. 122, 1555–1565. 10.1152/jn.00719.2018 31314634

[B33] Myers-Smith I. H. Grabowski M. M. Thomas H. J. Angers-Blondin S. Daskalova G. N. Bjorkman A. D.. (2019). Eighteen years of ecological monitoring reveals multiple lines of evidence for tundra vegetation change. Ecol. Monogr. 89, e01351. 10.1002/ecm.1351

[B34] Neuenschwander B. Capkun-Niggli G. Branson M. Spiegelhalter D. J. (2010). Summarizing historical information on controls in clinical trials. Clin. Trials 7, 5–18. 10.1177/1740774509356002 20156954

[B35] Palestro J. J. Bahg G. Sederberg P. B. Lu Z.-L. Steyvers M. Turner B. M. (2018). A tutorial on joint models of neural and behavioral measures of cognition. J. Math. Psychol. 84, 20–48. 10.1016/j.jmp.2018.03.003

[B36] Pariyadath V. Eagleman D. (2007). The effect of predictability on subjective duration. PLoS ONE 2, e1264. 10.1371/journal.pone.0001264 18043760 PMC2082074

[B37] Pastore M. Calcagnì A. (2019). Measuring distribution similarities between samples: a distribution-free overlapping index. Front. Psychol. 10, 1089. 10.3389/fpsyg.2019.01089 31231264 PMC6558420

[B38] Pelli D. G. Farell B. (1995). Psychophysical methods. Handbook Optics 1, 29–21.

[B39] Picconi F. Ryan C. Russo B. Ciotti S. Pepe A. Menduni M.. (2022). The evaluation of tactile dysfunction in the hand in type 1 diabetes: a novel method based on haptics. Acta Diabetol. 59, 1073–1082. 10.1007/s00592-022-01903-1 35641837 PMC9242965

[B40] Plummer M. (2003). “Jags: a program for analysis of bayesian graphical models using gibbs sampling,” in Proceedings of the 3rd International Workshop on Distributed Statistical Computing, Vol. 124 (Vienna), 1–10.

[B41] Plummer M. (2017). Jags Version 4.3. 0 User Manual [computer software manual]. Available online at: sourceforge.net/projects/mcmc-jags/files/Manuals/4.x2

[B42] Prins N. (2016). Psychophysics: A Practical Introduction. Cambridge, MA: Academic Press.

[B43] Prins N. Kingdom F. A. (2018). Applying the model-comparison approach to test specific research hypotheses in psychophysical research using the palamedes toolbox. Front. Psychol. 9, 1250. 10.3389/fpsyg.2018.01250 30083122 PMC6064978

[B44] Rouder J. N. Lu J. (2005). An introduction to bayesian hierarchical models with an application in the theory of signal detection. Psychon. Bull. Rev. 12, 573–604. 10.3758/BF03196750 16447374

[B45] Rouder J. N. Sun D. Speckman P. L. Lu J. Zhou D. (2003). A hierarchical bayesian statistical framework for response time distributions. Psychometrika 68, 589–606. 10.1007/BF0229561430998031

[B46] Ryan C. P. Bettelani G. C. Ciotti S. Parise C. Moscatelli A. Bianchi M. (2021). The interaction between motion and texture in the sense of touch. J. Neurophysiol. 126, 1375–1390. 10.1152/jn.00583.2020 34495782

[B47] Ryan C. P. Ciotti S. Cosentino L. Ernst M. O. Lacquaniti F. Moscatelli A. (2022). Masking vibrations and contact force affect the discrimination of slip motion speed in touch. IEEE Trans. Haptics 15, 693–704. 10.1109/TOH.2022.3209072 36149999

[B48] Schütt H. H. Harmeling S. Macke J. H. Wichmann F. A. (2016). Painfree and accurate bayesian estimation of psychometric functions for (potentially) overdispersed data. Vision Res. 122, 105–123. 10.1016/j.visres.2016.02.002 27013261

[B49] Steele F. Goldstein H. (2006). “12 multilevel models in psychometrics,” in Psychometrics, Volume 26 of Handbook of Statistics, eds C. Rao and S. Sinharay (Amsterdam: Elsevier), 401–420.

[B50] Stroup W. W. (2012). Generalized Linear Mixed Models: Modern Concepts, Methods and Applications. Boca Raton, FL: CRC Press.

[B51] Van de Schoot R. Winter S. D. Ryan O. Zondervan-Zwijnenburg M. Depaoli S. (2017). A systematic review of bayesian articles in psychology: the last 25 years. Psychol. Methods 22, 217. 10.1037/met0000100 28594224

[B52] van Rosmalen J. Dejardin D. van Norden Y. Löwenberg B. Lesaffre E. (2018). Including historical data in the analysis of clinical trials: Is it worth the effort? Stat. Methods Med. Res. 27, 3167–3182. 10.1177/0962280217694506 28322129 PMC6176344

[B53] Vehtari A. Gelman A. Gabry J. (2017). Practical bayesian model evaluation using leave-one-out cross-validation and waic. Stat. Comput. 27, 1413–1432. 10.1007/s11222-016-9696-4

[B54] Vehtari A. Gelman A. Simpson D. Carpenter B. Bürkner P.-C. (2021). Rank-normalization, folding, and localization: an improved r for assessing convergence of mcmc (with discussion). Bayesian Anal. 16, 667–718. 10.1214/20-BA1221

[B55] Wang X. Bradlow E. T. Wainer H. (2002). A general bayesian model for testlets: Theory and applications. Appl. Psychol. Meas. 26, 109–128. 10.1177/0146621602026001007

[B56] Wasserman L. (2000). Bayesian model selection and model averaging. J. Math. Psychol. 44, 92–107. 10.1006/jmps.1999.1278 10733859

[B57] Zhan P. Jiao H. Man K. Wang L. (2019). Using jags for bayesian cognitive diagnosis modeling: a tutorial. J. Educ. Behav. Stat. 44, 473–503. 10.3102/107699861982604027193366

[B58] Zhao Y. Staudenmayer J. Coull B. A. Wand M. P. (2006). General design bayesian generalized linear mixed models. Statist. Sci. 21, 35–51. 10.1214/088342306000000015

[B59] Zitzmann S. Lüdtke O. Robitzsch A. (2015). A bayesian approach to more stable estimates of group-level effects in contextual studies. Multivariate Behav. Res. 50, 688–705. 10.1080/00273171.2015.1090899 26717127

