# Supplementary material for: Bayesian hierarchical models and prior elicitation for fitting psychometric functions
Source: Front Comput Neurosci. 2023 Mar 2;17:1108311. doi: 10.3389/fncom.2023.1108311 (PMC10018033; doi:10.3389/fncom.2023.1108311)
Supplement: Supplementary file 1 [file Table_1.pdf]

Supplementary Material to: Bayesian Hierarchical Models and Prior Elicitation for Fitting Psychometric Functions

1 SUPPLEMENTARY TABLES

	Estimate	SE	z-value	p-value
α (intercept)	-2.02	0.14	-14.38	< 0.001
β (speed)	0.24	0.016	14.82	< 0.001
$\alpha_{vibr=32}$ (32 Hz)	0.38	0.09	4.00	< 0.001
$\beta_{vibr=32}$ (speed: 32 Hz)	-0.04	0.01	-4.10	< 0.001

Table S1. Fixed-effect parameters of GLMM for control experiment with fine-textured surface. From (Dallmann et al., 2015). The model is described in 3.1. The estimates of intercept and slope with masking vibrations at 32 Hz are equal to $\alpha + \alpha_{vibr=32}$ and to $\beta + \beta_{vibr=32}$, respectively.

	Masking 32 Hz	Estimate	95% CI	Width CI
PSE	No	8.57	(8.31, 8.82)	0.51
PSE	Yes	8.42	(8.12, 8.71)	0.59

Table S2. PSE from GLMM for control experiment with fine-textured surface. From (Dallmann et al., 2015). The model is described in 3.1

	Estimate	95% Credible Intervals	Width Cr Int
a^0 (intercept)	-1.97	(-2.11, -1.84)	0.27
b^0 (speed)	0.23	(0.21, 0.25)	0.04
a^1 (intercept 32 Hz)	-1.59	(-1.72, -1.4)	0.32
b^1 (speed 32 Hz)	0.19	(0.17, 0.21)	0.04

Table S3. Bayesian Posterior Estimates of parameters of the probit model as defined in Eq. (9)-(18), with corresponding 95% credible intervals. Experiment in 3.1

	Masking 32 Hz	Estimate	95% Credible Intervals	Width Cr Int
PSE	No	8.56	(8.33, 8.82)	0.49
PSE	Yes	8.41	(8.13, 8.68)	0.55

Table S4. Bayesian Posterior Estimates of PSE with 95% Credible Intervals, as defined Eq. (19)-(28). Experiment in 3.1

Parameter	Group	Masking	Estimate	SE	z-value	p-value
α^0 (intercept)	Control	No	-2.71	0.191	-14.13	< 0.001
α^0_2	Mild	No	0.262	0.258	1.02	0.31
α^0_3	Moderate	No	0.845	0.248	3.401	< 0.001
β^0 (slope)	Control	No	0.769	0.057	13.561	< 0.01
β^0_2	Mild	No	-0.108	0.077	-1.4	0.161
β^0_3	Moderate	No	-0.269	0.075	-3.61	< 0.01
α^1 (intercept)	Control	Yes	-1.99	0.149	-13.34	< 0.001
α^1_2	Mild	Yes	0.245	0.206	1.190	0.234
α^1_3	Masking	Yes	0.505	0.203	2.493	0.013
β^1 (slope)	Control	Yes	0.520	0.0421	12.36	< 0.001
β^1_2	Mild	Yes	-0.063	0.058	-1.072	0.29
β^1_3	Moderate	Yes	-0.15	0.0581	-2.59	0.01

Table S5. Fixed-effect parameters of GLMM in Experiment in 3.2. P-values were computed with the Wald test. To simplify the reading of the model, differently from Picconi et al. (2022), we fit the GLMM separately in the two masking vibration conditions.

Parameter	Group	Masking	Estimate	Inferior	Superior
intercept	Controls	0	-2.64	-2.84	-2.50
intercept	Mild	0	-2.36	-2.65	-2.13
intercept	Moderate	0	-1.81	-2.11	-1.58
intercept	Controls	1	-1.99	-2.23	-1.82
intercept	Mild	1	-1.71	-1.98	-1.48
intercept	Moderate	1	-1.50	-1.78	-1.25
slope	Controls	0	0.75	0.70	0.80
slope	Mild	0	0.64	0.56	0.72
slope	Moderate	0	0.48	0.42	0.56
slope	Controls	1	0.52	0.47	0.59
slope	Mild	1	0.45	0.38	0.53
slope	Moderate	1	0.37	0.31	0.45
pse	Controls	0	3.54	3.38	3.70
pse	Mild	0	3.71	3.54	3.88
pse	Moderate	0	3.76	3.57	3.95
pse	Controls	1	3.84	3.66	4.04
pse	Mild	1	3.81	3.59	4.04
pse	Moderate	1	4.02	3.80	4.28

Table S6. GLMM Estimation of the parameters with 95% Bootstrap Confidence Intervals. Data-set in 3.2

	Posterior Estimate	95% Credible Intervals	Width Cr Int
b^0_{ctrl}	0.74	(0.65, 0.85)	0.2
b^0_2	0.62	(0.54, 0.72)	0.18
b^0_3	0.49	(0.39, 0.6)	0.21
b^1_{ctrl}	0.5	(0.44, 0.56)	0.12
b^1_2	0.46	(0.36, 0.56)	0.2
b^1_3	0.36	(0.30, 0.41)	0.11
PSE^0_{ctrl}	3.54	(3.41, 3.68)	0.27
PSE^0_2	3.7	(3.54, 3.9)	0.36
PSE^0_3	3.7	(3.53, 3.89)	0.56
PSE^1_{ctrl}	3.84	(3.65, 4.02)	0.37
PSE^1_2	3.78	(3.59, 3.99)	0.4
PSE^1_3	4.04	(3.8, 4.26)	0.46

Table S7. Bayesian posterior estimates - Data-set analyzed with the Bayesian model described in 3.2

REFERENCES

Dallmann, C. J., Ernst, M. O., and Moscatelli, A. (2015). The role of vibration in tactile speed perception. *Journal of neurophysiology* 114, 3131–3139

Picconi, F., Ryan, C., Russo, B., Ciotti, S., Pepe, A., Menduni, M., et al. (2022). The evaluation of tactile dysfunction in the hand in type 1 diabetes: a novel method based on haptics. *Acta Diabetologica* , 1–10
